# Development and Functional Characterization of a Versatile Radio-/Immunotheranostic Tool for Prostate Cancer Management

**DOI:** 10.3390/cancers14081996

**Published:** 2022-04-14

**Authors:** Claudia Arndt, Ralf Bergmann, Franziska Striese, Keresztély Merkel, Domokos Máthé, Liliana R. Loureiro, Nicola Mitwasi, Alexandra Kegler, Frederick Fasslrinner, Karla Elizabeth González Soto, Christin Neuber, Nicole Berndt, Noemi Kovács, David Szöllősi, Nikolett Hegedűs, Gyula Tóth, Jan-Philipp Emmermann, Kuzhuvelil B. Harikumar, Tibor Kovacs, Michael Bachmann, Anja Feldmann

**Affiliations:** 1 Helmholtz-Zentrum Dresden-Rossendorf, Institute of Radiopharmaceutical Cancer Research, D-01328 Dresden, Germany; c.arndt@hzdr.de (C.A.); r.bergmann@hzdr.de (R.B.); f.striese@rotop-pharmaka.de (F.S.); l.loureiro@hzdr.de (L.R.L.); n.mitwasi@hzdr.de (N.M.); a.kegler@hzdr.de (A.K.); k.gonzalez@hzdr.de (K.E.G.S.); c.neuber@hzdr.de (C.N.); n.berndt@hzdr.de (N.B.); a.feldmann@hzdr.de (A.F.); 2 Mildred Scheel Early Career Center, Faculty of Medicine Carl Gustav Carus, TU Dresden, D-01328 Dresden, Germany; frederick.fasslrinner@uniklinikum-dresden.de; 3 Department of Biophysics and Radiation Biology, Faculty of Medicine, Semmelweis University, Tűzoltó u. 37-47, H-1094 Budapest, Hungary; merkel.keresztely@phd.semmelweis-univ.hu (K.M.); domokos.mathe@hcemm.eu (D.M.); noemi.kovacs@hcemm.eu (N.K.); szollosi.david@med.semmelweis-uni.hu (D.S.); hegedus.nikolett@med.semmelweis-univ.hu (N.H.); jan.emmermann@stud.semmelweis.hu (J.-P.E.); 4 University No. 1 Clinic for Surgery, Faculty of Medicine, Semmelweis University, H-1094 Budapest, Hungary; 5 Hungarian Centre of Excellence for Molecular Medicine, In Vivo Imaging Advanced Core Facility, H-6723 Szeged, Hungary; 6 Medical Clinic and Polyclinic I, Medical Faculty, University Hospital Carl Gustav Carus, Technical University Dresden, D-01307 Dresden, Germany; 7 Department: Cyclotron & Radiochemistry, Pozitron Ltd., H-1094 Budapest, Hungary; gyula.toth@pet.hu; 8 Cancer Research Program, Rajiv Gandhi Centre for Biotechnology (RGCB), Thiruvananthapuram 695 014, Kerala, India; harikumar@rgcb.res.in; 9 Institute of Radiochemistry and Radioecology, University of Pannonia, H-8200 Veszprem, Hungary; kt@almos.uni-pannon.hu; 10 National Center for Tumor Diseases (NCT), D-01307 Dresden, Germany; German Cancer Research Center (DKFZ), D-69120 Heidelberg, Germany; Faculty of Medicine and University Hospital Carl Gustav Carus, Technische Universität Dresden, D-01307 Dresden, Germany; Helmholtz-Zentrum Dresden-Rossendorf (HZDR), D-01328 Dresden, Germany; 11 German Cancer Consortium (DKTK), Partner Site Dresden and German Cancer Research Center (DKFZ), D-69120 Heidelberg, Germany; 12 Tumor Immunology, University Cancer Center (UCC), University Hospital Carl Gustav Carus, Technical University Dresden, D-01307 Dresden, Germany

**Keywords:** prostate cancer, PSCA, PSMA, IgG4, CAR T cell, theranostics, Ac-225, Cu-64, (18)F-JK-PSMA-7

## Abstract

**Simple Summary:**

In previous studies, we described a modular Chimeric Antigen Receptor (CAR) T cell platform which we termed UniCAR. In contrast to conventional CARs, the interaction of UniCAR T cells does not occur directly between the CAR T cell and the tumor cell but is mediated via bispecific adaptor molecules so-called target modules (TMs). Here we present the development and functional characterization of a novel IgG4-based TM, directed to the tumor-associated antigen (TAA) prostate stem cell antigen (PSCA), which is overexpressed in prostate cancer (PCa). We show that this anti-PSCA IgG4-TM cannot only be used for (i) redirection of UniCAR T cells to PCa cells but also for (ii) positron emission tomography (PET) imaging, and (iii) alpha particle-based endoradiotherapy. For radiolabeling, the anti-PSCA IgG4-TM was conjugated with the chelator DOTAGA. PET imaging was performed using the ^64^Cu-labeled anti-PSCA IgG4-TM. According to PET imaging, the anti-PSCA IgG4-TM accumulates with high contrast in the PSCA-positive tumors of experimental mice without visible uptake in other organs. For endoradiotherapy the anti-PSCA IgG4-TM-DOTAGA conjugate was labeled with ^225^Ac^3+^. Targeted alpha therapy resulted in tumor control over 60 days after a single injection of the ^225^Ac-labeled TM. The favorable pharmacological profile of the anti-PSCA IgG4-TM, and its usage for (i) imaging, (ii) targeted alpha therapy, and (iii) UniCAR T cell immunotherapy underlines the promising radio-/immunotheranostic capabilities for the diagnostic imaging and treatment of PCa.

**Abstract:**

Due to its overexpression on the surface of prostate cancer (PCa) cells, the prostate stem cell antigen (PSCA) is a potential target for PCa diagnosis and therapy. Here we describe the development and functional characterization of a novel IgG4-based anti-PSCA antibody (Ab) derivative (anti-PSCA IgG4-TM) that is conjugated with the chelator DOTAGA. The anti-PSCA IgG4-TM represents a multimodal immunotheranostic compound that can be used (i) as a target module (TM) for UniCAR T cell-based immunotherapy, (ii) for diagnostic positron emission tomography (PET) imaging, and (iii) targeted alpha therapy. Cross-linkage of UniCAR T cells and PSCA-positive tumor cells via the anti-PSCA IgG4-TM results in efficient tumor cell lysis both in vitro and in vivo. After radiolabeling with ^64^Cu^2+^, the anti-PSCA IgG4-TM was successfully applied for high contrast PET imaging. In a PCa mouse model, it showed specific accumulation in PSCA-expressing tumors, while no uptake in other organs was observed. Additionally, the DOTAGA-conjugated anti-PSCA IgG4-TM was radiolabeled with ^225^Ac^3+^ and applied for targeted alpha therapy. A single injection of the ^225^Ac-labeled anti-PSCA IgG4-TM was able to significantly control tumor growth in experimental mice. Overall, the novel anti-PSCA IgG4-TM represents an attractive first member of a novel group of radio-/immunotheranostics that allows diagnostic imaging, endoradiotherapy, and CAR T cell immunotherapy.

## 1. Introduction

While localized prostate cancer (PCa) is well manageable, treatment options for metastatic disease are still limited [1]. In the last decade, significant progress has been especially made towards PCa theranostics aiming to design antibody (Ab)- or small molecule-based radiopharmaceuticals for both tumor imaging and targeted endoradiotherapy [2]. Despite advances in imaging with [^68^Ga]Ga-PSMA-11 or [^18^F]F-DCFPyl for PCa detection/staging [3,4,5,6], and encouraging results achieved with novel radiotherapeutic agents, such as the bone-targeting radionuclide radium-223 [7] or [^177^Lu]Lu-PSMA-617 [8], eventually all patients with advanced disease will progress to metastatic castration-resistant prostate cancer (mCRPC). The low five-year survival rate of 30%, together with a median survival of only 10 to 21.7 months [1,9,10], underline that new treatment options for high-risk mCRPC patients are urgently needed.

An emerging therapeutic modality comprises targeted immunotherapies with chimeric antigen receptors (CARs) T cells that revolutionized in particular the therapeutic landscape of hematologic malignancies [11,12,13], and thus hold also great promise for solid tumor treatment. However, successful clinical translation towards solid cancers faces some obstacles, as reflected by clinical results with both prostate stem cell antigen (PSCA)-specific (NCT03873805, NCT02744287, NCT03198052) and prostate-specific membrane-antigen (PSMA)-specific CAR T cells (e.g., NCT01140373, NCT01140373, NCT03089203, NCT04053062), which are rather suboptimal in efficacy. In particular, CAR T cells have to overcome an immunologically cold tumor microenvironment (TME) [14] and efficiently traffic to and infiltrate distinct tumor sites that are located in the bones in >80% of cases [15]. Moreover, all PCa-specific tumor-associated antigens (TAAs) described so far are also expressed on healthy tissues, increasing the risk for on-target/off-tumor effects. Taken together, CAR T cell therapies must adapt to the challenging nature of PCa to augment their efficacy and safety in patients. One possibility to achieve this goal is the use of adaptor CAR platforms [16], which provide the required flexibility.

In our group, we have established the switchable CAR platform “UniCAR” [17,18,19,20,21,22]. In contrast to conventional CAR T cells, UniCAR T cells do not recognize a surface molecule but bind a short peptide epitope (UniCAR epitope) [23,24,25,26,27]. Therefore, cross-linkage of UniCAR T cells with target cells and thus anti-tumor activity is mediated via the target module (TM) carrying the UniCAR epitope and a tumor-specific binding domain [28,29,30]. With regard to management of adverse reactions including on-target/off-tumor effects, the UniCAR system offers the possibility of therapy control via the separated TMs. As shown by several preclinical and a first clinical study (NCT04230265) [31], permanent infusion of TMs in a tumor patient turns anti-tumor activity of UniCAR T cells “ON”. After elimination of the TM from the body, UniCAR T cells automatically return to a “switch OFF” mode [32,33,34,35,36,37].

Besides the problem of steering, the UniCAR platform may also overcome further challenges in PCa therapy by repurposing TMs as theranostic compounds. In this regard, TMs can be labeled with radionuclides suitable for positron emission tomography (PET) or single photon emission computed tomography (SPECT) and used for diagnostic imaging of the primary tumor and metastases prior and during therapy [20]. This would allow not only the staging of the disease but also the assessment of the response to therapy. Based on the acquired information, more accurate treatment decisions can be taken, e.g., whether the treatment must and can be extended. Alternatively, TMs could be radiolabeled with a therapeutic radionuclide such as lutetium-177 or actinium-225 for targeted radioimmunotherapy. Besides their own therapeutic effects, such TMs should increase local inflammation, which might help to attract and activate immune effector cells, including CAR T cells. Altogether, the application of one molecule for diagnosis, endoradiotherapy, and CAR T cell therapy could lead to a novel, beneficial, combinatorial cancer therapy option for PCa patients.

To show first proof-of-concept for this idea, we developed a TM for the UniCAR system with pharmacological features that allow its specific accumulation at the tumor site for both diagnostic PET imaging and radioimmunotherapy. For this purpose, a novel recombinant IgG4-based TM directed against the PSCA was constructed. The novel anti-PSCA IgG4-TM was functionalized with the chelator DOTAGA for radiolabeling with either the PET radionuclide ^64^Cu or the therapeutic radionuclide ^225^Ac. In this study, we evaluated its use for UniCAR T cell therapy, after radiolabeling for diagnostic imaging, as well as targeted alpha therapy (TAT).

## 2. Materials and Methods

### 2.1. Materials

If not noted otherwise, all commercially obtained chemicals were of analytical grade or better and purchased from Sigma-Aldrich (Merck KGaA, Darmstadt, Germany). Aqueous solutions were prepared using ultrapure water (resistivity, 18.2 MΩ·cm). Buffers used for coupling reactions or radiolabeling were purified from metal contamination using Chelex 100 resin (Bio-Rad Laboratories GmbH, Feldkirchen, Germany) and filtered through a 0.22 μm filter (Millipore, Merck KGaA, Darmstadt, Germany). The bifunctional p-NCS-Bz-DOTAGA (C_27_H_38_N_60_O_9_S; Mw = 622.69 g mol^−1^; Molecular Weight), the chelator 1,4,7,10-tetraazacyclododecane-1,4,7,10-tetraacetic acid (DOTA), diethylenetriaminepenta-acetic acid (DTPA), and ethylenediaminetetraacetic acid (EDTA) were purchased from CheMatech (Dijon, France). All reagents used in cell culture experiments were purchased from Biological Industries (Sartorius Stedim Biotech GmbH, Goettingen, Germany). Actinium-225 was obtained from ITG Isotope Technologies Garching GmbH (Munich, Germany).

### 2.2. Cell Lines

All cell lines were obtained from American Type Culture Collection (ATCC, Manassas, VA, USA). The PCa cell lines PC3 and LNCaP were genetically modified to overexpress human PSCA according to a previously published protocol [34]. PC3 wildtype (wt), LNCaP-PSCA, and PC3-PSCA cells were used for in vitro experiments. For in vivo studies, PC3-PSCA tumor cells were further genetically engineered via lentiviral transduction to overexpress the genes encoding firefly luciferase and PSMA (see [34]). In all in vivo experiments, the resulting PC3-PSCA/PSMA Luc+ cell line was transplanted. Besides the original PCa cell line, all genetically modified PCa cell lines were authenticated by genetic genotyping (ATCC). Tumor cell lines or Ab-producing 3T3 cell lines were cultured in RPMI complete media [38] or DMEM complete media [38], respectively. All cell lines were incubated at 37 °C with 5% CO_2_.

### 2.3. Generation and Cultivation of UniCAR T Cells

T cells were isolated from buffy coats of healthy donors (German Red Cross, Dresden, Germany) by density gradient centrifugation and subsequent magnetic isolation via the Pan T Cell Isolation Kit (Miltenyi Biotech GmbH, Bergisch Gladbach, Germany). The study received approval by the institutional review board of the Faculty of Medicine of the TU Dresden (EK138042014). T cells were maintained in RPMI complete medium with 50 U/mL human IL-2 (Miltenyi Biotec GmbH) until UniCAR T cell production. Lentiviral transduction of human T cells was performed as described previously [39,40]. Following activation with T Cell TransAct™ (Miltenyi Biotec GmbH), T cells were infected 2–3 times with lentiviral particles encoding for the UniCAR. During genetic modification and subsequent expansion, T cells were kept in TexMACS™ medium (Miltenyi Biotec GmbH) supplemented with human IL-2, human IL-7, and human IL-15 (all Miltenyi Biotec GmbH). Transduction efficiency was monitored by expression of the co-translated EGFP marker protein using flow cytometry. Experiments were conducted with unsorted UniCAR T cells that were cultured without cytokines for 24 h.

### 2.4. Cloning of Recombinant Antibodies

All recombinant PSCA-specific antibodies (Abs) were generated based on the sequences of the fully human anti-PSCA IgG1 monoclonal antibody (mAb) Ha1-4.121 given in the patents EP 2,428,522 A1 and US 8,013,128 B2. To clone the single-chain fragment variables (scFvs) anti-PSCA Ha1-4.121c.5 and anti-PSCA Ha1-4.121c.26, V_L_c.5 or V_L_c.26, respectively, were fused in silico to V_H_ via a flexible peptide linker ((G_4_S)_2_-GASAA-(G_4_S)_2_) in V_L_-V_H_ orientation. For secretion into the cell culture supernatant, the scFvs were N-terminally linked to the murine Ig kappa leader sequence. At the C-terminus of the scFvs, we fused the UniCAR epitope (E5B9). Downstream of the UniCAR epitope, we added a myc- and hexahistidine (His)-tag for Ab purification and detection. The scFv sequences were ordered from Eurofins Genomics (Ebersbach, Germany) and subsequently cloned into the lentiviral expression vector p6NST50 via the restriction sites *Xba*I and *KspA*I.

After verification of the anti-PSCA reactivity, the functional anti-PSCA scFv sequence was fused to an IgG4-Ab backbone according to previously published constructs [36,41]. For this purpose, the anti-PSCA scFv Ha1-4.121c.26 was amplified by PCR using the primers scFvHa1-4.121c.26-SfiI for (5′-GGCCCAGCCGGCCGGTTCCGACATCGTCATG-3′) and scFvHa1-4.121c.26-MreI rev (5′-CGCCGGCGCAGAGCTCACTGTCACGAG-3′) to insert the restriction sites *Sfi*I and *Mre*I. The PCR product was subcloned in pGEMTeasy vector (Promega GmbH, Mannheim, Germany) and subsequently inserted into the lentiviral expression vector p6NST50_huIgG4-Fc via *Sfi*I/*Mre*I restriction sites. Finally, the UniCAR epitope E5B9 and a His-tag were introduced at the 3′ end of the PSCA-IgG4-Fc sequence. Therefore, a double-stranded DNA sequence with 5′ and 3′ overhangs compatible with *Xba*I and *KspA*I restriction sites was prepared by annealing the oligos E5B9-His for (5′-ctagaGGCGGCGGAGGGTCTGCAGCTGCCAAACCTCTGCCCGAAGTGACAGACGA-GTATGGGCCAGGCGGTGGTGGAAGCCACCATCATCACCACCATTGA-3′) and E5B9-His rev (5′-TCAATGGTGGTGATGATGGTGGCTTCCACCACCGCCTGGCCCAT-ACTCGTCTGTCACTTCGGGCAGAGGTTTGGCAGCTGCAGACCCTCCGCCGCCT 3′). Using Thermo Scientific™ Buffer R (Thermo Fisher Scientific GmbH, Schwerte, Germany) as reaction buffer, 200 pmol of each of the oligos were incubated at 95 °C for 5 min. After cooling the reaction mixture for 15 min at room temperature, a 1:10 dilution of the annealed oligos was used for ligation into the vector p6NST50_PSCA-huIgG4-Fc via *Xba*I/*KspA*I restriction sites. All restriction enzymes and buffers were purchased from Thermo Fisher Scientific GmbH (Schwerte, Germany).

### 2.5. Expression, Purification, and Biochemical Characterization of Ab Constructs

To generate permanent Ab-producing cell lines, murine 3T3 cells were modified via lentiviral transduction [38,42] with the lentiviral vectors encoding the anti-PSCA scFv Ha1-4.121c.5 (Mw = 31 kDa), anti-PSCA scFv Ha1-4.121c.26 (Mw = 32 kDa) or anti-PSCA IgG4-TM (Mw = 112 kDa). Purification of recombinant Abs from cell culture supernatants was performed according to already described procedures: The scFvs were purified by Ni-NTA affinity chromatography [38], whereas the purification of the anti-PSCA IgG4-TM was achieved via protein A affinity chromatography [41]. After dialysis against 1 × PBS, purity and concentration of recombinant proteins were determined in the elution fractions by SDS-PAGE under reducing conditions and subsequent staining with Coomassie Brilliant Blue G250 [43] or Quick Coomassie^®^ Stain (Serva, Heidelberg, Germany). For immunoblotting, proteins were transferred to nitrocellulose membranes and detected via the C-terminal His-tag as previously described [38,42]. Dimerization of the anti-PSCA IgG4-TM was further analyzed via size exclusion high-performance liquid chromatography (SE-HPLC) as detailed in Albert et al. [44].

### 2.6. Flow Cytometric Binding Analysis

Binding properties and affinities of the novel PSCA-specific recombinant Abs were assessed by flow cytometry according to previously published protocols [45]. Briefly, PC3-PSCA tumor cells were incubated with recombinant Abs at indicated concentrations for 1 h at 4 °C. Ab binding was either detected via the His- or E5B9-tag using a PE-labeled anti-His Ab (Miltenyi Biotec GmbH) or 10 μg/mL of the anti-La (5B9) mAb [46], in combination with the Goat anti-mouse IgG Fc Cross-Adsorbed Secondary Ab, PE (Thermo Fisher Scientific GmbH) or PE Goat anti-mouse IgG (minimal x-reactivity) Ab (Biolegend, San Diego, CA, USA), respectively. Stained cells were analyzed with MACSQuant Analyzer 10 and MACSQuantifiy Software (Miltenyi Biotec GmbH).

### 2.7. Activation Assay and Enzyme-Linked Immunosorbent Assay (ELISA)

5 × 10^4^ UniCAR T cells were incubated alone or in the presence of 1 × 10^4^ tumor cells in a 96-well U-bottom plate. Recombinant Abs were added at a concentration of 5 nM. After 24 h, cell-free supernatant was harvested and analyzed for secreted cytokines by ELISA as previously described [45]. Human TNF ELISA Set, Human IFN-Gamma ELISA Set, Human IL-2 ELISA Set, and BD OptEIA Reagent Set B were purchased from BD Biosciences (Becton Dickinson GmbH, Heidelberg, Germany) and used according to the manufacturer’s instructions.

After 24 h of co-cultivation, UniCAR T cells were further examined with respect to their activation status. For this purpose, cells of one triplicate were pooled and stained with anti-human CD3-PE-Vio^®^770 or CD3-VioBlue and anti-human CD69-APC Abs (all Miltenyi Biotec GmbH) for 15 min at 4 °C. To allow exclusion of dead cells during analysis, counterstaining with 1 μg/mL propidium iode/PBS solution was performed. Flow cytometric data were acquired with the MACSQuant Analyzer 10 (Miltenyi Biotec GmbH).

### 2.8. Chromium Release Assay

Standard chromium release assays were conducted as described in detail by Feldmann and colleagues [38]. In brief, 5 × 10^3^ chromium-labeled tumor cells were incubated with UniCAR T cells at an effector-to-target cell (E:T) ratio of 5:1 in the presence or absence 5 nM or decreasing concentrations of recombinant Ab. After 24 h, radioactivity in co-culture supernatants was measured with a MicroBeta Microplate Counter (PerkinElmer LAS GmbH, Rodgau, Germany).

### 2.9. Radiolabeling of the TM with ^64^Cu and ^225^Ac

All solvents were purchased from Sigma-Aldrich, Merck KGaA (Darmstadt, Germany). The anti-PSCA IgG4-TM was conjugated with a 10 molar excess of p-NCS-Bz-DOTAGA (C_27_H_38_N_60_O_9_S; Mw = 622.69 g mol^−1^; molecular weight 622.69) in 0.1 M borate buffer (pH 8.5) at 4 °C for 12 h, resulting in the anti-PSCA IgG4-TM conjugated with DOTAGA (DOTAGA-TM). Then, it was washed 4 times with PBS via spin filtration at 4 °C using Amicon Ultra-0.5 centrifugal filter devices with a molecular weight cutoff of 50 K (Amicon Ultra 3 K 1 device, Merck-Millipore, Merck KGaA, Darmstadt, Germany).

The no carrier added (n.c.a.) [^64^Cu]Cu^2+^ was produced at the Helmholtz-Zentrum Dresden-Rossendorf on a TR-Flex (Advanced Cyclotron Systems Inc., Richmond, BC, Canada) by ^64^Ni(p,n)^64^Cu nuclear reaction and prepared as reported previously [47]. For radiolabeling of the DOTAGA-TM with ^64^Cu, [^64^Cu]CuCl_2_ (200 MBq in 0.01 M HCl, 0.3 M NH_4_OAc, pH 5.0) was added to 0.2 nmol (22.4 µg) of the DOTAGA-TMs and incubated at 38 °C for 30 min. The [^64^Cu]Cu-DOTAGA-TM is referred to as ^64^Cu-TM. The reaction mixture was cooled to 20 °C, quenched with 10 mM DTPA (10 μL) to complex any free or nonspecifically bound ^64^Cu^2+^, and purified via spin filtration as described. Labeling yield and radiochemical purity were determined using radio instant thin-layer chromatography (radio-ITLC). The radiolabeled conjugate was retained at the origin, Rf = 0.0, while unbound radioactivity moved with the solvent (10 mM sodium citrate and 0.1 mM EDTA, Rf = 0.8–1.0). The developed chromatograms were analyzed by autoradiography using an in vivo Multispectral Imaging System (Bruker Daltonik GmbH, Bremen, Germany) [48].

^225^Ac (1 MBq) in 0.01 M HCl was added to 0.5 M ammonium acetate buffer (pH 5.2), followed by 0.2 nmol (22.4 µg) of DOTAGA−TM conjugate. The mixture was incubated at 38 °C for 4 h, cooled to 20 °C, quenched with 10 mM EDTA (10 μL) to complex any free or nonspecifically bound ^225^Ac, and finally purified via spin filtration as described. For comparison, DOTA (25 μg) was mixed with ^225^Ac in 200 μL of ammonium acetate buffer and heated for 20 min at 90 °C. Hereafter, [^225^Ac]Ac-DOTAGA-TM is referred to as ^225^Ac-TM. For quality control, radio-ITLC of the ^225^Ac-TM and the ^225^Ac-DOTA was carried out on SG stripes (Merck KGaA, Darmstadt, Germany) using 0.1 M citrate, 0.01 M EDTA. After development, the chromatography strip was stored for at least 1 h until radiochemical equilibrium was obtained between ^225^Ac (half-life [T_1⁄2_], 9.9 d) and its daughter nuclide ^221^Fr (T_1/2_ 4.8 min). The percentage of complexed ^225^Ac was determined by ITLC on silica gel-impregnated glass fiber sheets (Agilent Technologies Deutschland GmbH, Waldbronn, Germany) using 0.1 M citrate buffer at pH 4.0. The radiolabeled conjugate was retained at the origin, Rf = 0.0, while unbound radioactivity and the [^225^Ac]Ac-DOTA moved with the solvent, Rf = 0.8–1.0.

### 2.10. ^18^F-JK-PSMA-7 Synthesis

^18^F-JK-PSMA-7 is a fluorine-18 labeled ligand developed by the FZ-Jülich and the University of Cologne [49]. It was synthetized in a simplified one step automated method by a direct radiofluorination of (S)-2-[3-((S)1-Carboxy-5-((6-trimethylammonium-2-methoxypyridine-3-carbonyl)-amino)-pentyl)-ureido]-pentanedioic acid trifluoroacetate salt (1) as a precursor compound. The precursor was purchased from Trasis S.A. (Ans, Belgium), while the ^18^F fluoride was produced via the ^18^O(p,n)^18^F nuclear reaction through proton irradiation of enriched (98%) ^18^O water (ROTEM, Germany) using an Eclipse HP cyclotron (Siemens Healthcare GmbH, Erlangen, Germany).

Briefly, the radiosynthesis was carried out by utilizing a cassette-based AllInOne synthesizer with built in radio-HPLC, conducted by a synthesis sequence developed by Trasis S.A. (Ans, Belgium). The cassettes and reagent kits necessary for the radiosynthesis of ^18^F-JK-PSMA-7 were purchased ready-to-use from Trasis S.A. as well. The irradiated ^18^O water was passed through an ion exchange cartridge, where the ^18^F- was trapped, then it was eluted with a water/acetonitrile solution of tetrabutyl ammonium bicarbonate. Afterwards, the solution was evaporated to dryness, and the trimethyl ammonium salt precursor (1) dissolved in acetonitrile was added. The fluoride anion displaced the trimethyl ammonium triflate leaving group in a SNAr reaction, resulting in the fluorinated protected intermediate (2). The crude compound of ^18^F-JK-PSMA 7 was obtained by acidic hydrolysis of the intermediate at elevated temperature carried out in the same reactor vessel by the addition of ortho phosphoric acid. To obtain nearly 100% radiochemically pure product, semi-preparative HPLC purification was performed by injecting the whole volume of the reaction mixture onto a XBridge BEH Shield RP18 5 μm 10 × 250 mm (Waters Corporation, Milford, MA, USA) HPLC column. The pure radiopharmaceutical (3) was eluted with a diluted phosphoric acid to acetonitrile mixture ratio of 80:20. The HPLC eluent was removed by solid phase extraction of ^18^F-JK-PSMA-7 on a C18 SepPak cartridge followed by saline wash and elution with a small amount of ethanol. The residual radioactivity in the tubing was collected by rinsing with isotonic saline into a vial containing sodium ascorbate. After filtration through a 0.2 μm sterile Supor Membrane filter (Pall GmbH, Dreieich, Germany), the ^18^F-JK-PSMA-7 solution was ready for i.v. injection.

### 2.11. Animals, Feeding, Husbandry, Preparation, and Animal Experiments

Animals were allowed free access to food and water and maintained under temperature, humidity, and light-controlled conditions. Tumor size and body weight were regularly measured.

To analyze the immunotherapeutic potential of the anti-PSCA IgG4-TM, eight week old, male, NXG-immunodeficient mice (NOD-*Prkdc^scid^-IL2rg^Tm1^*/Rj, JANVIER LABS, Le Genest-Saint, France) were used. Mice were divided into three groups of five animals each. Animals of Group 1 were subcutaneously injected into the right thigh with 1 × 10^6^ PC3-PSCA/PSMA cells. Group 2 received a mixture of 1 × 10^6^ PC3-PSCA/PSMA cells and 1 × 10^6^ UniCAR T cells, while 100 pmol TM/mouse was additionally applied subcutaneously to the treatment group (Group 3). All mixtures were adjusted to a total volume of 100 µL per mouse (in PBS). Tumor growth was monitored over two days using the reporter luciferase, overexpressed by PC3-PSCA/PSMA Luc+ cells. Ten minutes prior to the optical imaging, mice were anesthetized [34,44] and injected intraperitoneally with 150 µL XenoLight D-Luciferin Potassium Salt (15 mg/mL) (PerkinElmer LAS GmbH, Rodgau, Germany). Detection of luminescence signal was performed using the In Vivo Xtreme Imaging System (Bruker, Bremen, Germany). For luminescence imaging, exposure times were set to 10 min. Data were analyzed with the MI 5.3 and MS 1.3 software (Bruker, Bremen, Germany).

For TAT and PET imaging, five week old, male, Rj:NMRI-Foxn1^nu/nu^ mice (mutant outbred mouse with thymic aplasia causing a T cell deficiency; JANVIER LABS, Le Genest-Saint-Isle, France) were subcutaneously injected into the right flank with 2 × 10^6^ PC3-PSCA/PSMA Luc+ tumor cells. Four weeks after inoculation of the tumor cells, TAT experiments were started. At this time, the tumors had reached a diameter of 100–400 mm^3^. The xenografted mice were divided into two groups each of five mice. The five control mice were injected with 0.04 nmol DOTAGA-TM (4.5 µg per 100 µL PBS) i.v. via tail vein. In the treatment group, mice were injected with 5 kBq (135 nCi) of ^225^Ac-TM (0.04 nmol, 4.5 µg DOTAGA-TM). Animals were sacrificed at day 60 or before that time point when they appeared to suffer, in accordance with animal welfare regulations.

The effect of the TAT on tumor response was assessed by comparison of the Specific Growth Rates (SGR). Tumor diameters were measured twice a week using a caliper. The tumor volume was calculated for each time point as *π/6·a·b^2^*, where *a* is the longest and *b* is the perpendicular shorter tumor diameter. Additionally, after the PET measurements the animal bed with the anesthetized mice was translated to the CT and a whole-body CT was measured. From the data sets, the tumors were delineated with software package ROVER (ABX GmbH, Dresden, Germany) and the volumes calculated.

Tumor growth kinetics were evaluated from the growth curves of individual tumors. Starting point was the time of injection of DOTAGA-TM (control) or the ^225^Ac-TM. The tumor growth kinetics were evaluated by an equation that describes the growth with a constant doubling time (DT) *Y = Y_0_·exp(k·t). Y_0_* is the *Y* value when *t* (time) is zero. It is expressed in the same units as *Y*; *k* is the rate constant, expressed in reciprocal of the *t* axis time units. If *t* is in days, then *k* is expressed in inverse days. *k* is equal to the SGR. The DT is calculated as *DT = ln(2)/SGR* [50]. The tumor SGR were compared by an unpaired *t*-test. Statistical significance was determined using the Holm-Sidak method, with alpha = 0.05, without assuming a consistent SD. The specific tumor volumes, calculated as *Y/Y_0_*, were also compared for each time point with *t*-test. The statistical calculations were performed using GraphPad Prism version 7.00 for Mac OS X, GraphPad Software (La Jolla, CA, USA.)

### 2.12. ^64^Cu-TM Positron Emission Tomography

Anesthetized, spontaneously breathing animals were allowed to stabilize for 10 min after preparation. The animals were positioned on a heated bed to maintain the body temperature at 37 °C. The PET studies were carried out with a microPET P4^®^ (Siemens Healthcare GmbH, Erlangen, Germany). The activity of the injection solution was measured in a well counter (Isomed 2000, Dresden, Germany) cross-calibrated to the PET scanners. The PET acquisition of 120 min emission scan was started, and the infusion of 15 MBq (0.4 mCi) ^64^Cu-TM was initiated with a delay of 10 s. An amount of 0.1 mL of solutions of ^64^Cu-TM were infused over 1 min (with a Harvard apparatus 44 syringe pump) into a lateral tail vein. Additional PET scans were carried out over one hour, after 31 h and 44 h.

### 2.13. ^18^F-JK-PSMA-7 Positron Emission Tomography

The ^18^F-JK-PSMA-7 was synthetized at Pozitron Ltd. Budapest Hungary, (Dept: Cyclotron & Radiochemistry). The xenotransplanted tumor mice (five animals per group) were imaged with ^18^F-JK-PSMA-7 at day 43 after the start of the treatment with the ^225^Ac-TM. The mice were injected i.v. with 10 MBq (0.27 mCi) of ^18^F-JK-PSMA-7. After 30 min the mice were anesthetized with isofluran/oxygen and positioned on the animal bed of the microPET P4^®^. The measurement was continued over 30 min. The images were reconstructed with a 3D OSEM MAP algorithm. Data were evaluated using ROVER (ABX GmbH, Dresden, Germany).

### 2.14. X-ray CT Imaging

The animals undergoing ^18^F-JK-PSMA-7 PET imaging were also imaged in a Mediso nanoX-CT^TM^ (Mediso Medical Imaging Systems Ltd., Budapest, Hungary), a dedicated small animal X-ray system, with helical whole body scanning of 360 projections and 45 kV of source voltage. Animals were positioned in the same anesthesia and temperature maintaining bed during the same imaging session with PET. CT images were reconstructed using the built-in filtered back projection algorithm of the device and coregistered to PET using ROVER.

### 2.15. Statistical Analysis

Values are expressed as mean ± SEM and were compared using ANOVA or the unpaired Student’s *t*-test with Welch’s correction and an F-test to compare the variances (GraphPad Prism 7.0 from GraphPad Software, La Jolla, CA, USA). The Kruskal–Wallis test was performed to identify the differences between groups. All statistical testing was performed using GraphPad Prism 7.0 software. Significant difference was set at * *p* < 0.05; ** *p* < 0.01; *** *p* < 0.001.

## 3. Results

### 3.1. Antibody Preparation and Characterization

**Figure 1:**
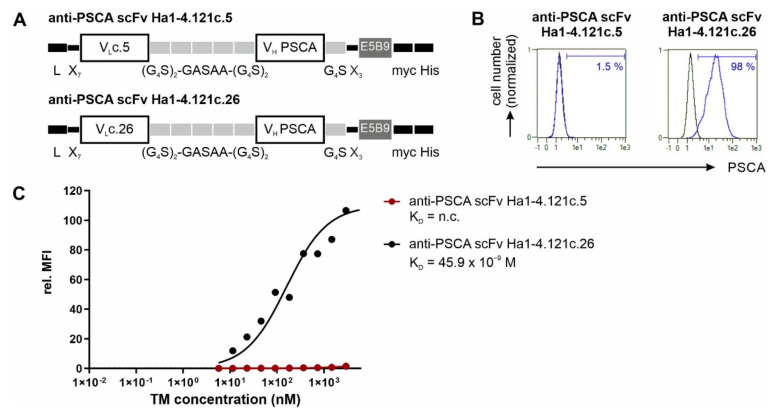
Functional characterization of V_L_c.5 and V_L_c.26 of the human anti-PSCA IgG1 Ab Ha1-4.121. (**A**) Schematic representation of the anti-PSCA scFvs Ha1-4.121 containing either the V_L_c.5 or V_L_c.26. C-terminally recombinant Abs were equipped with the E5B9-, myc- and hexahistidine-tag (His-tag). L, leader peptide; V_L_, variable domain of the light chain; V_H_, variable domain of the heavy chain; scFv, single-chain fragment variable. (**B**,**C**) Flow cytometry data of anti-PSCA scFv Ha1-4.121c.5 and anti-PSCA scFv Ha1-4.121c.26 using PC3-PSCA cells. After incubation of tumor cells with (**B**) 20 ng/µL or (**C**) increasing scFv concentrations, the binding of the respective scFv was detected via primary anti-La(5B9) mAb, and secondary goat anti-mouse IgG-PE Ab. (**B**) Histograms show percentage of stained cells (blue graphs) in comparison to the respective negative controls (black graphs). (**C**) For calculation of the apparent affinity of the scFvs towards PSCA, Ab concentrations were plotted against the relative mean fluorescence intensity (rel. MFI) values. Results of one representative experiment is shown; n.c., not calculable.

For construction of the theranostic anti-PSCA IgG4-TM, we selected the fully human anti-PSCA IgG1 Ab Ha1-4.121. The sequences of this Ab were taken from the patents EP 2,428,522 A1 and US 8,013,128 B2. Based on the published sequences, we reconstructed the variable domains of the heavy (V_H_) and light (V_L_) chains. Unfortunately, the hybridoma expresses two V_L_ genes (V_L_c.5 and V_L_c.26) (see patents EP 2,428,522 A1, US 8,013,128 B2). Therefore, it remained unclear if both or only one of the V_L_ in combination with the identified V_H_ encode a functional, PSCA-specific Ab-binding domain. For this reason, both the V_L_c.5 or V_L_c.26 domains were recombinantly fused with the common Ha1-4.121 V_H_ domain via flexible glycine–serine linkers to obtain the two scFvs, anti-PSCA Ha1-4.121c.5 and anti-PSCA Ha1-4.121c.26 (Figure 1A). Subsequently, both scFvs were eukaryotically expressed (data not shown) and compared regarding their binding activity towards PSCA (Figure 1B,C). As shown by flow cytometry analysis (Figure 1B,C), the anti-PSCA scFv Ha1-4.121c.26 was able to specifically bind PC3-PSCA cells with high affinity (K_D_ = 45.9 nM), while the anti-PSCA scFv Ha1-4.121c.5 showed no binding towards PSCA-expressing target cells. According to these data, the V_L_c.26 domain is the actual and functional V_L_ domain of the human anti-PSCA Ab Ha1-4.121. Consequently, the V_L_c.26 domain was further used for development of the novel anti-PSCA IgG4-TM.

**Figure 2:**
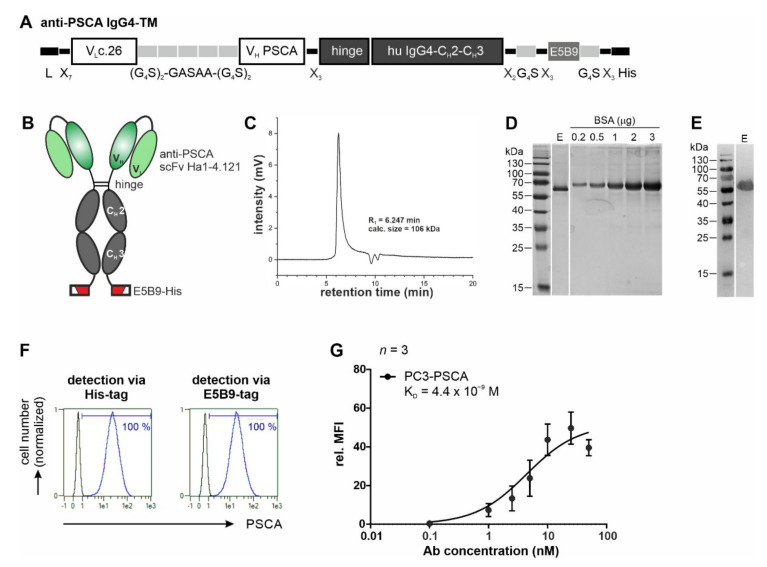
Biochemical characterization and binding properties of the recombinant anti-PSCA IgG4-TM. (**A**) The anti-PSCA scFv Ha1-4.1.2c.26 was fused to the hinge and Fc-region of human IgG4 molecules via flexible peptide linkers. In addition, the E5B9- and His-tag were fused to the C-terminus of the recombinant Ab. (**B**) The anti-PSCA IgG4-TM forms homodimers that are covalently connected via disulfide bounds in the hinge region. L, leader peptide; V_L_, variable domain of the light chain; V_H_, variable domain of the heavy chain; C_H_, constant domain of the heavy chain; scFv, single-chain fragment variable. (**C**–**E**) After purification via protein A affinity chromatography, the elution fraction (**E**) containing the anti-PSCA IgG4-TM was analyzed via (**C**) SE-HPLC and (**D**,**E**) SDS-PAGE. (**D**) Proteins (TM monomer, Mw = 56 kDa) separated via SDS-PAGE were stained via Quick Coomassie^®^ Stain solution to determine TM purity and concentration by means of a BSA standard. (**E**) After Western blotting, the anti-PSCA IgG4-TM was detected via its C-terminal His-tag. Uncropped WB in Figure S1. (**F**,**G**) Binding activity of the anti-PSCA IgG4-TM was evaluated by flow cytometry. PC3-PSCA cells were incubated with (**F**) 20 ng/µL or (**G**) increasing TM concentrations. Binding was detected via anti-His-PE Ab or primary anti-La(5B9) mAb, and secondary PE goat anti-mouse IgG (minimal x-reactivity) Ab. (**F**) Histograms show percentage of stained cells (blue graphs) in comparison to the respective negative controls (black graphs). (**G**) For calculation of affinity towards PSCA, Ab concentrations were plotted against the relative mean fluorescence intensity (rel. MFI) values. Results of three independent experiments are shown (rel. MFI ± SEM).

To generate the multimodal anti-PSCA IgG4-TM, the V_L_c.26 and V_H_ of the human anti-PSCA Ab Ha1-4.121 were connected to the hinge and Fc domain (C_H_2 and C_H_3) of human IgG4 molecules, respectively. For immunotherapeutic application and detection, the Ab construct was additionally equipped with the UniCAR epitope E5B9 [51] and a His-tag (Figure 2A,B). For expression of the resulting anti-PSCA IgG4-TM, a permanent 3T3 production cell line was established. The recombinant PSCA-specific TM was successfully purified from cell culture supernatants of this production cell line via protein A affinity chromatography with high yield and purity (Figure 2C–E). Due to the presence of cysteine residues in the hinge region, the protein should be able to form a dimer stabilized by disulfide bonds resulting in the formation of homodimers with a theoretical molecular weight of 112 kDa. Under reducing conditions, the homodimer should separate into single polypeptide chains with an expected size of 56 kDa. Indeed, comparison of the native molecular weight obtained by SE-HPLC (Figure 2C) with the molecular weight obtained by SDS-PAGE under reducing conditions (Figure 2D,E) confirms that the native anti-PSCA IgG4-TM exists as a homodimer.

In a next step, binding properties were analyzed by flow cytometry using PC3-PSCA cells. As shown in Figure 2F, the anti-PSCA IgG4-TM specifically binds to PSCA-overexpressing PCa cells. Binding was confirmed via both the E5B9- and His-tag. With an estimated apparent K_D_ value of 4.4 nM, the bivalent anti-PSCA IgG4-TM had a 10-fold increased affinity/avidity (Figure 2G) towards PSCA when compared to the monovalent anti-PSCA scFv Ha1-4.121c.26 (Figure 1C).

### 3.2. UniCAR T Cell Immunotherapy with the Anti-PSCA IgG4-TM

**Figure 3:**
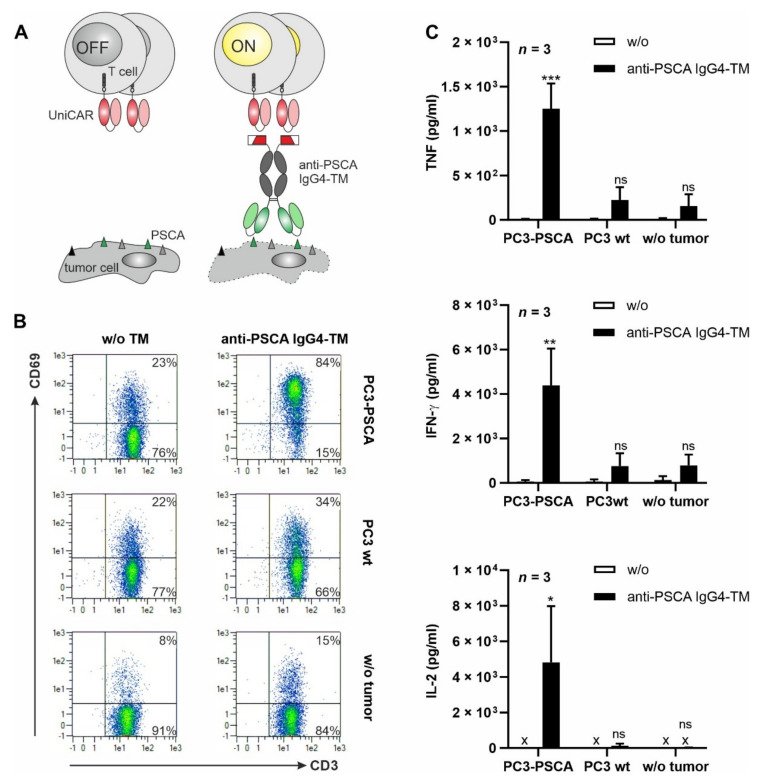
TM-mediated cross-linkage with tumor cells results in UniCAR T cell activation and cytokine release. (**A**) In the UniCAR T cell approach, T cells are engineered to express a universal chimeric antigen receptor (UniCAR) that recognizes the E5B9 peptide of the nuclear antigen La/SS-B. Thus, under physiological conditions UniCAR T cells are switched “OFF”. To engage the killing capabilities of UniCAR T cells against tumor cells (“ON”), an E5B9-tagged target module (TM) recognizing a tumor-associated antigen is required. (**B**,**C**) UniCAR T cells were incubated alone or with PC3-PSCA or PC3 wt cells at an E:T ratio of 5:1 in the presence or absence of 5 nM anti-PSCA IgG4-TM. After 24 h, (**B**) CD69 expression on UniCAR T cells and (**C**) secretion of TNF, IFN-γ and IL-2 were examined via flow cytometry or ELISA (x: not detectable), respectively. (**B**) Data of one representative experiment with one T cell donor are shown. (**C**) Summarized data of three different T cell donors are shown as mean ± SEM. (* *p* < 0.05, ** *p* < 0.01, *** *p* < 0.001 compared to samples w/o the anti-PSCA IgG4-TM; two-way ANOVA with post-hoc Šídák’s multiple comparisons test).

Due to incorporation of the E5B9-tag, the anti-PSCA IgG4-TM carries all typical features of a TM that can be used in the well-established UniCAR approach [51]. As summarized in Figure 3A, the UniCAR system comprises two main components: (i) T cells modified to express UniCARs and (ii) tumor-specific TMs (here directed against PSCA). As UniCAR T cells specifically recognize and bind to the E5B9 peptide epitope [51], E5B9-tagged TMs can function as bridging molecules between tumor and T cells. This TM-mediated cross-linkage finally results in efficient tumor cell lysis.

**Figure 4:**
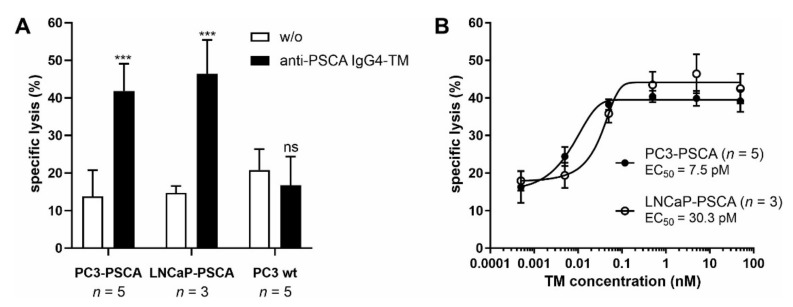
Redirection of UniCAR T cells for tumor cell killing via the novel anti-PSCA IgG4-TM. In 24 h standard chromium release assays, UniCAR T cells were incubated with PC3-PSCA, LNCaP-PSCA, or PC3 wt cells at an E:T ratio of 5:1 in the presence or absence of either (**A**) 5 nM or (**B**) decreasing concentrations of the anti-PSCA IgG4-TM. Mean specific lysis ± SEM of three or five different T cell donors are shown (*** *p* < 0.001 compared to samples w/o the anti-PSCA IgG4-TM; two-way ANOVA with post-hoc Šídák’s multiple comparisons test).

To prove that the novel anti-PSCA IgG4-TM can be utilized as a TM in the UniCAR system, various co-cultivation assays were conducted. UniCAR T cells were incubated with PSCA-positive or PSCA-negative tumor cells in the presence or absence of the novel TM. As exemplified by the upregulation of the early activation marker CD69, UniCAR T cells were activated in a target-specific and strictly TM-dependent manner (Figure 3B and Figure S2A). Upon anti-PSCA IgG4-TM mediated cross-linkage with PC3-PSCA tumor cells, UniCAR T cells were engaged for significant secretion of the pro-inflammatory cytokines TNF and IFN-γ, as well as the growth-promoting cytokine IL-2 (Figure 3C and Figure S2B ). As shown in Figure 4A, the TM-mediated cross-linkage of tumor and UniCAR T cells finally resulted in specific tumor cell lysis. Neither in absence of tumor cells nor in the presence of PSCA-negative PC3 wt cells, an unspecific release of cytokines or tumor cell killing could be observed. As depicted in Figure 4B, the anti-PSCA IgG4-TM successfully engaged UniCAR T cells for efficient tumor cell killing with an EC_50_ (half-maximal effective concentration) value of 7.5 or 30.3 pM for PC3-PSCA or LNCaP-PSCA cells, respectively. According to previously published data, the anti-PSCA IgG4-TM showed comparable killing efficiency to other PCa-specific or IgG-based TMs [34,36,41,52].

**Figure 5:**
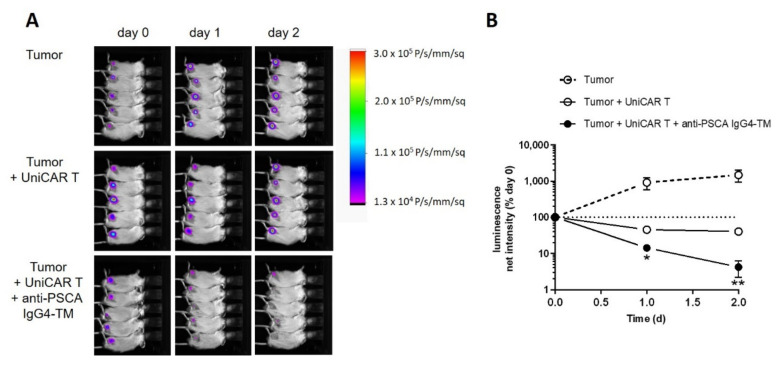
Evaluation of the immunotherapeutic potential of the anti-PSCA IgG4-TM in experimental mice. Immunodeficient NXG mice were subcutaneously injected with PC3-PSCA/PSMA Luc+ alone or in combination with UniCAR T cells in the absence or presence of the anti-PSCA IgG4-TM. (**A**) Luminescence images for each mouse after day 0, day 1, and day 2 are shown. (**B**) Quantitative analysis of luminescence signals. For each mouse, net intensities (P/s/mm^2^) were normalized to net intensities measured at day 0. Mean luminescence net intensities ± SEM for five mice per group are shown (* *p* < 0.05, ** *p* < 0.01, compared to the control group “Tumor + UniCAR T”, Student’s *t*-test).

In vivo functionality of the anti-PSCA IgG4-TM within the UniCAR system was analyzed in an immunodeficient mouse model. For this purpose, animals were subcutaneously injected with mixtures of (i) PC3-PSCA/PSMA Luc+ tumor cells, (ii) PC3-PSCA/PSMA Luc+ tumor cells plus UniCAR T cells, or (iii) PC3-PSCA/PSMA Luc+ tumor cells, UniCAR T cells, and TM. As summarized in Figure 5, the anti-PSCA IgG4-TM efficiently activated UniCAR T cells for tumor cell killing in mice. According to the luciferase signal, tumors significantly regressed when compared to the control groups and were not detectable in three out of five mice already after two days. Differences in tumor growth (not statistically significant) between control Group 1 (tumor) and control Group 2 (tumor + UniCAR T cells), can be most likely attributed to T cell-dependent rejection reactions against alloantigens expressed on tumor cells. Such effects are strongly donor-dependent and can be even more pronounced in mice due to introduction of human (genetically modified) T cells into a murine environment.

### 3.3. Radiolabeling of the Anti-PSCA IgG4-TM with Cu-64 and Ac-225

Having proven the efficacy of the novel anti-PSCA IgG4-TM for UniCAR T cell immunotherapy, its suitability for diagnostic tumor imaging and endoradiotherapy was evaluated. For this purpose, the TM was modified with DOTAGA and subsequently labeled with either ^64^Cu as a diagnostic radionuclide or ^225^Ac as a therapeutic radionuclide. Both the diagnostic ^64^Cu-TM and the therapeutic ^225^Ac-TM could be synthetized with comparable radiochemical purity (>97%) according to the ITLC. The ^64^Cu-TM had a radiochemical purity of 97.1% with a molar activity of 2.7 GBq/µmol (9 TBq/g). The ^225^Ac-TM showed a radiochemical purity of 97.6% (one experiment) and a molar activity of 0.019 GBq/µmol (0.17 GBq/g).

### 3.4. Imaging with ^64^Cu-TM in Xenotransplanted Mice

**Figure 6:**
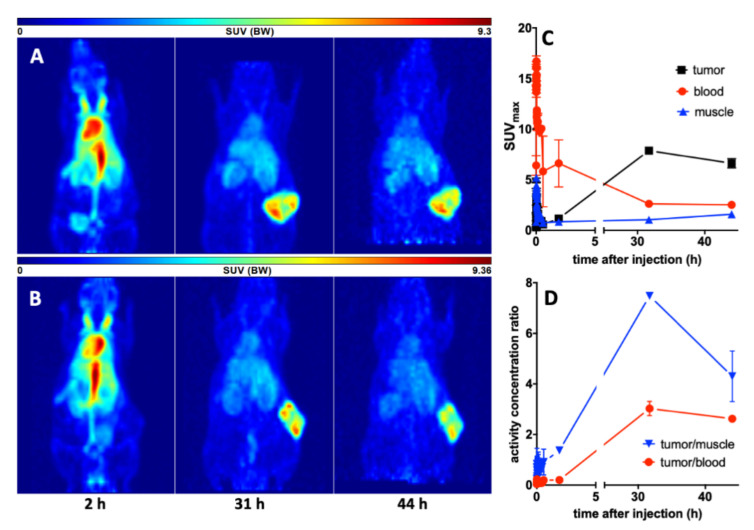
Imaging and kinetics of the ^64^Cu-TM distribution in PC3-PSCA/PSMA Luc+ tumor bearing NMRI-Foxn1^nu/nu^ mice. Maximum intensity projections of the PET studies of (**A**) mouse #1 and (**B**) mouse #2 at 2, 31, and 44 h after injection of the ^64^Cu-TM. (**C**) Activity–concentration–time curves in the tumor, blood, and muscle. (**D**) Tumor-to-muscle and tumor-to-blood ratios. Values are means ± SEM of two animals.

First, we tested the ability of the TM-DOTAGA conjugate to target PSCA-expressing tumors in vivo. Therefore, PC3-PSCA/PSMA Luc+ tumor-bearing mice were injected with 15 MBq ^64^Cu-TM, which is equivalent to 3 MBq of a pure positron-emitting isotope. This relatively large amount allowed PET imaging over two days with good statistics. As shown in Figure 6, the ^64^Cu-labeled anti-PSCA IgG4-TM is localized to and is extensively retained in the PC3-PSCA/PSMA-Luc+ tumors. The activity distribution was evaluated as a function of time (0–2, 31, 44 h) (see Figure 6). In the distribution phase, the ^64^Cu-TM cleared from the blood with a biological half-life of 6.0 h converting to a calculated plateau of 32.5% of the starting activity concentration. The maximal activity concentrations in the tumors were reached after 31 h. At this time, the tumors could be clearly delineated from the background in the PET images. This can be attributed to the high number of PC3-PSCA/PSMA-Luc+ cells being targeted in the tumors. In the first phase (0–2 h), the heart and the venous blood vessel system were clearly visible. The high activity at this time in the neck region was caused from the venae jugularis. At later time points, only the circulation and highly perfused organs such as heart, liver, kidneys, and the tumor were visible. No evidence of any ^64^Cu-TM accumulation in the salivary glands was found.

### 3.5. Targeted Alpha Therapy with the ^225^Ac-TM in a Prostate Cancer Mouse Model

#### 3.5.1. Tumor Growth Delay after ^225^Ac-TM Treatment

**Figure 7:**
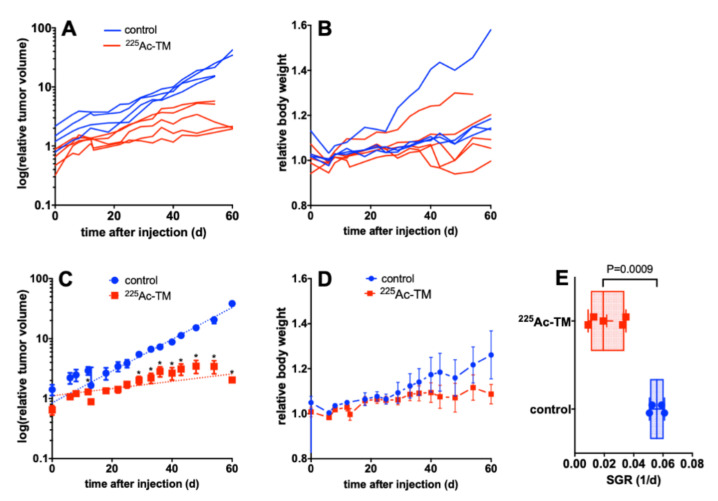
Targeted alpha therapy with the ^225^Ac-TM in a xenograft mouse model of PCa. (**A**,**B**) Individual or (**C**,**D**) averaged curves for (**A**,**C**) the tumor volume, and (**B**,**D**) body weight changes after DOTAGA-TM (control, *n* = 4) or ^225^Ac-TM (*n* = 5) injection in tumor-bearing mice. (**E**) The SGR of the relative tumor volumes were calculated with the exponential growth equation. SGR values for tumors of the control (0.0558 ± 0.0025, *n* = 4) and treatment group (0.0217 ± 0.0052, *n* = 5) correspond to doubling times of 12.4 and 32.0 days, respectively. Data are presented as means ± SEM. For each time point, the SGR of the single animals was analyzed with an unpaired *t*-test without assumption of consistent SD; the calculations were corrected for multiple comparisons using Holm-Sidak method; alpha of 0.05 was defined as ‘statistically significant’.

In a next step, we examined whether the novel anti-PSCA IgG4-TM could be easily repurposed as a tool for TAT. Therefore, NMRI-Foxn1^nu/nu^ mice were subcutaneously injected with PC3-PSCA/PSMA Luc+ tumor cells. When tumors reached 100–400 mm^3^ in size, mice were treated with either DOTAGA-TM (control) or ^225^Ac-TM. In comparison to the control group, the ^225^Ac-TM showed significant potency against PC3-PSCA/PSMA-Luc+ tumors in a xenograft mouse model (Figure 7). At day 43 post-treatment, tumor growth was significantly reduced (*p* = 0.05) in the therapy group (Figure 7A,C).

The SGR of the relative tumor volumes calculated with the exponential growth equation were significantly (*p* = 0.0009) different with 0.0558 ± 0.0025 (*n* = 4) for the control animals and 0.0217 ± 0.0052 (*n* = 5) for the treatment group, corresponding to doubling times of 12.4 and 29 days, respectively. The body weights of the animals of both groups (Figure 7B,D) didn’t differ over the observation time.

As mice were injected with a low amount of activity (5 kBq ^225^Ac-TM/animal corresponding to 0.15 µg TM/mouse or 1.3 pmol TM/mouse), the visualization of ^225^Ac-TM gamma-emitting isotopes in the mice by SPECT was not possible [51]. However, the biodistribution and kinetics of the ^225^Ac-TM is expected to be very close to the biodistribution of the ^64^Cu-TM.

#### 3.5.2. Imaging of the Tumors with ^18^F-JK-PSMA-7

**Figure 8:**
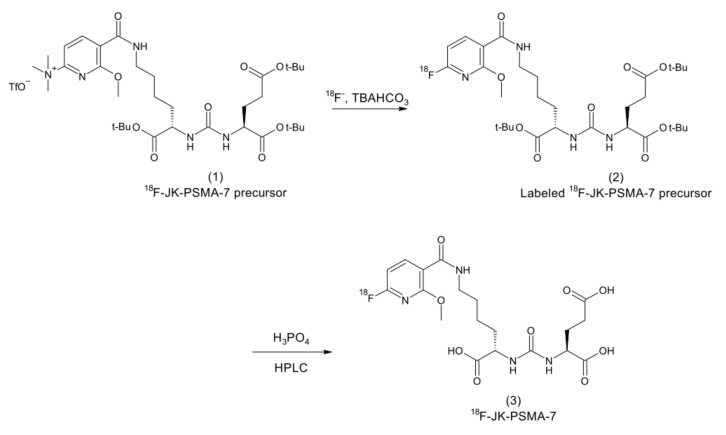
Scheme of the radiosynthesis of ^18^F-JK-PSMA-7.

To (i) identify living tumor cells, (ii) to get an independent metabolic response parameter of the ^225^Ac-TM-based alpha therapy, and (iii) to show the radiotracer distribution in the tumors, animals of the TAT study (see Figure 6) were additionally imaged with the PSMA targeting compound ^18^F-JK-PSMA-7. ^18^F-JK-PSMA-7 is a fluorine-18 labeled PSMA ligand [49]. Thus, it can bind to PSMA, which is also overexpressed on the selected model cell line PC3-PSCA/PSMA Luc+. ^18^F-JK-PSMA-7 was synthetized using a simplified one-step automated method by a direct radiofluorination of (S)-2-[3-((S)1-Carboxy-5-((6-trimethylammonium-2-methoxypyridine-3-carbonyl)-amino)-pentyl)-ureido]-pentanedioic acid trifluoroacetate salt (1) as a precursor compound (Figure 8). The non–decay-corrected radiochemical yield of ^18^F-JK-PSMA-7 was 20% after isolation by HPLC.

**Figure 9:**
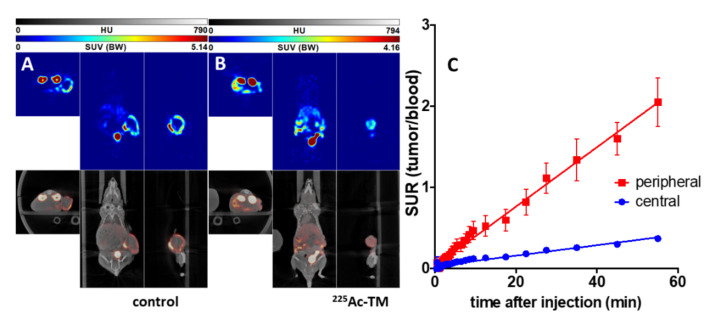
^18^F-JK-PSMA-7 imaging of mice after targeted alpha therapy with the ^225^Ac-TM. Orthogonal sections of PET/CT studies of a representative (**A**) control and (**B**) ^225^Ac-TM-treated mouse with xenotransplanted PC3-PSCA/PSMA Luc+ tumors are shown. The PET studies were carried out at day 43 after treatment start and 2 h after single intravenous injection of 10 MBq ^18^F-JK-PSMA-7 with an imaging duration of 30 min. (**C**) Kinetics of the ^18^F-JK-PSMA-7 in the xenotransplanted untreated PC3-PSCA/PSMA Luc+ tumors (control) expressed as tumor uptake ratio (tumor-to-blood standard uptake ratio, SUR) (mean ± SEM of two animals of the control group) in the periphery (peripheral) and central part (central) of the tumors.

The ^18^F-JK-PSMA-7 accumulation in the PC3-PSCA/PSMA-Luc+ tumors was evaluated on day 43 after treatment start in the control and ^225^Ac-TM-treated animals (Figure 9). The tumors were clearly delineated. Time–activity curves were exemplarily analyzed for two animals and are shown for peripheral and central tumor areas. The peripheral tumor part accumulated approximately five times more ^18^F-JK-PSMA-7 than the central part. The other activity localizations in the mice correlate to the normal distribution of this imaging agent known from humans, except for the accumulation in the kidneys. In one animal of each group, a lymph node or small metastasis became visible.

**Figure 10:**
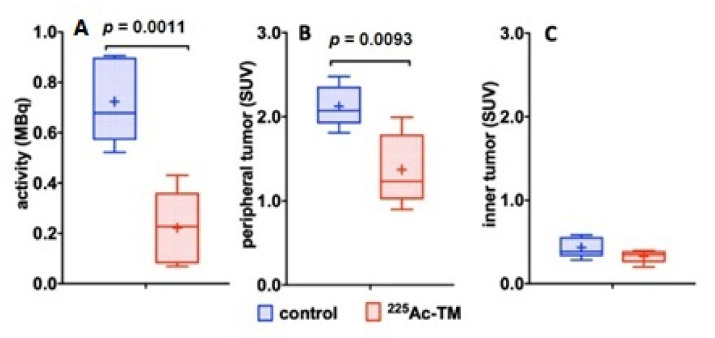
Quantitative comparison of the ^18^F-JK-PSMA-7 activity distribution. (**A**) ^18^F-JK-PSMA-7 activity amounts in the total tumor (MBq), (**B**) activity concentrations (SUV) in the periphery of the tumors, and (**C**) in the central part of the tumors are shown. The box and whiskers plots show mean SUV values for each group indicated as “+” in the control (*n* = 4) and ^225^Ac-TM treated group (*n* = 5).

The quantitative comparison of the ^18^F-activity distribution in the control and ^225^Ac-TM group showed a significant decrease of the activity amount (Figure 10A) in the total tumor, and a significant decrease of the activity concentration in the peripheral tumor (Figure 10B). The activity concentrations (SUV) in the central, low perfused, and necrotic part of the tumors (Figure 10C) did not differ between both groups.

## 4. Discussion

Despite multiple treatment options encompassing a variety of disciplines, late-stage mCRPC remains a difficult to treat disease. Along with advances of theranostic radiopharmaceuticals, the therapeutic landscape is currently being shaped by emerging immunotherapies. Non-invasive imaging techniques that accompany therapy play an important role in providing a patient-centered, timely optimized treatment regimen. Therefore, in this study, we attempted to combine the three main pillars of PCa management into one approach by using the flexible adaptor CAR platform UniCAR and described a novel IgG4-based TM for radio-/immunotheranostics of PCa. Considering that PSMA radiopharmaceuticals currently dominate the theranostic landscape but also have some limitations, particularly in patients with low or heterogeneous PSMA expression profiles [52,53], we developed a multimodal TM directed against an alternative PCa-specific target, PSCA, that could complement existing PSMA-directed theranostic strategies. PSCA is overexpressed in more than 80% of PCa tissues and in the majority of bone metastasis [54,55,56,57,58,59], while showing restricted expression in normal tissues [38,42,54,55,56,57,60]. Elevated PSCA levels further correlate with increased tumor stage, grade, and progression to androgen independence [38,42,54,55,56,57,60] making it interesting and suitable for Ab-based immunotherapy and imaging of late-stage prostate cancer. The here-described novel anti-PSCA IgG4-TM represents the molecular basis for novel multimodal PCa applications. It can be used (i) for PSCA-directed UniCAR T cell immunotherapy, and after radiolabeling, (ii) for diagnostic imaging or (iii) targeted endoradiotherapy.

Considering that the TM is administered multiple times for distinct applications in patients, a key criterion for Ab engineering was to minimize its immunogenicity. Monoclonal Abs or Ab fragments from foreign species were shown to trigger undesired anti-drug immune reactions in humans that may cause loss of therapeutic efficiency, altered pharmacokinetics, or even serious adverse reactions [61,62,63]. To circumvent this risk and allow repeated applications, the novel anti-PSCA IgG4-TM was designed based on the V_L_ and V_H_ of the fully human anti-PSCA mAb Ha1-4.121 and the Fc-domain of human IgG4 molecules. As IgG4 molecules show little or no binding to classical Fcγ receptors or complement C1q [64,65,66], unwanted activation of complement and antibody-dependent cellular cytotoxicity can be further limited. Owing to its high affinity for the neonatal Fc receptor [67], IgG4 retains the characteristic prolonged in vivo kinetics of IgG molecules [66].

With respect to UniCAR immunotherapy, the novel anti-PSCA IgG4-TM shows functionality and efficiency. UniCAR T cells were activated for significant secretion of pro-inflammatory cytokines and tumor cell killing both in vitro and in vivo in a highly TM-dependent and antigen-specific manner. As published for conventional CAR design, optimal synapse distances are critical for proper signaling and cytolytic activity of CAR T cells [68]. Altering the extracellular spacer length, and thus the space between tumor and target cells, can considerably influence CAR T cell effectiveness [68]. Despite its larger size and increased affinity, the anti-PSCA IgG4-TM allows the formation of a functional immune synapse and induces highly effective tumor cell lysis with an EC_50_ value of 7.5 or 30.3 pM. The TM-efficacy is thus comparable to previously described, smaller, PCa-specific TMs (EC_50_ = 12 pM), underlining the plasticity and flexibility of the UniCAR system for different TM formats [16,34]. The same observations could not only be made with previously developed GD2- or STn-specific, IgG4-based UniCAR TMs [18,32,36,41], but are also consistent with other adaptor CAR platforms successfully applying adaptor molecules of different size and pharmacokinetics (summarized in [16]). Moreover, we could demonstrate that simultaneous targeting of PSCA and PSMA is feasible by applying the anti-PSCA IgG4-TM together with the previously established PSMA-L TM [69] (see also Figure S3). The application of dual-targeting strategies in PCa patients might be important for future clinical translation, as it might help to overcome problems such as inter- and intra-patient heterogeneities and tumor escape due to antigen loss. In terms of imaging, a combination of both TMs might further improve PCa detection and monitoring of molecular responses to therapy as discussed above.

Theranostics in PCa commonly involves the consecutive application of different small molecule-based radiotracers for diagnostic imaging and endoradiotherapy [3]. If molecules with different coordination chemistry are used, pharmacokinetics may differ, possibly hampering the theranostic purpose [3]. To achieve comparable in vivo kinetics, small molecule inhibitors using one chelating moiety for both applications, such as MIP-1095 [70], PSMA I&T [71], or PSMA-617 [72], were developed. Accordingly, functionalization of the novel, multimodal anti-PSCA IgG4-TM also required the conjugation of one appropriate chelator that allows the complexation with both diagnostic and therapeutic radionuclides such as ^68^Ga, ^64^Cu, ^111^In, ^177^Lu, and ^225^Ac [73,74,75,76,77,78,79]. For PET-based imaging of PCa, we selected ^64^Cu. Due to its low positron-energy and relative long physical half-life, it seems to be a promising radionuclide for diagnostic imaging. In this study, the alpha-emitting radionuclide ^225^Ac was employed for TAT. Alpha-particles are potent therapeutic effectors when directed to cancer targets [80,81,82]. The short effective range (several cell diameters) and high particle energies (5–8 MeV) make ^225^Ac effective for targeting of small tumor lesions, including bone metastases commonly found in mCRPC patients. When compared to beta-emitters, alpha-particles show higher efficacy at lower off-target toxicity [82,83]. To enable labeling of the anti-PSCA IgG4-TM with both ^64^Cu and ^225^Ac, we selected the chelator DOTAGA. One difficulty in the application of this chelator is the high temperature necessary for radiolabeling. To circumvent this problem, a temperature of 38 °C was used in combination with extended incubation times for TM labeling. After removal of free and non-specifically bound radionuclides, the achieved molar activities for ^64^Cu and ^225^Ac were comparable to literature data [77,84,85,86]. Conjugation of DOTAGA to the anti-PSCA IgG4-TM does not compromise its PSCA binding, diagnostic imaging, or TAT effect.

PET experiments showed a high contrast tumor accumulation of the ^64^Cu-TM. The intact ^64^Cu-TM increasingly accumulated in the tumor with maximum enrichment after 31 h. Activity slowly cleared from the blood and after two days was only found in the blood and the tumor resulting in optimal target-to-background ratios that did not significantly change over time. The absence of activity accumulation in other organs, including excretory organs and the reticuloendothelial system, proves TM stability, absence of TM aggregates, and excretion of radioactive metabolites. Although the physical half-life of the ^64^Cu only allows imaging over two days, our data clearly underline suitability of the TM for high contrast PET imaging of PSCA-expressing PCa.

Pharmacokinetic properties of the anti-PSCA IgG4-TM also play an important role for UniCAR immunotherapy. When compared to previously described PCa-specific TMs [33,38,39,45,61,87], the anti-PSCA IgG4-TM has a considerably prolonged serum half-life. In clinical practice, this would allow reduction of TM infusions and could intensify elimination of residual PCa cells, which is particularly important in highly aggressive and rapidly progressing mCRPC. Although TM elimination from the blood is delayed, switchability of the UniCAR system remains as an important control mechanism, which is especially relevant to avoid long-term destruction of PSCA-expressing healthy tissues.

The extended serum half-life of the anti-PSCA IgG4-TM in combination with its stable tumor accumulation could enhance local tumor radiation, and thus its therapeutic effectiveness in the context of radioimmunotherapy. Half-life extension proved to be also advantageous for small molecule inhibitors, showing increased local irradiation [88]. The ^225^Ac-TM has demonstrated therapeutic efficiency in a PCa xenograft mouse model. Single injection of 5 kBq ^225^Ac-TM in tumor-bearing mice resulted in significant tumor control after 23 days until the end of the study. Complete tumor elimination was probably not achieved, as tumors were potentially too large at therapy initiation, limiting the effectiveness of alpha-emitters that possess uneven micro-distribution and a short effective range. Due to low ^225^Ac activity concentrations applied (5 kBq/animal), therapy accompanying SPECT imaging of the daughter radionuclide was not possible. Nonetheless, the exchange of ^64^Cu for ^225^Ac should not significantly affect the biodistribution, as this is dominated by the large size of the DOTAGA-TM (112 kDa).

In addition to determination of the relative tumor volumes, ^18^F-JK-PSMA-7 imaging was performed at day 43 to assess tumor viability and to confirm therapeutic effects. ^18^F-JK-PSMA-7 PET analysis revealed a typical distribution of the co-expressed marker PSMA on tumor cells. In line with tumor volume measurements, significantly larger total PSMA levels and thus tumor cell amounts were found in the control group when compared to the ^225^Ac-TM-treated group. The generally observed differences in ^18^F-JK-PSMA-7 activities between peripheral and internal tumor areas can be explained by an uneven perfusion of the tumors, whereby necrotic areas and internal interstitial pressure decrease radiotracer uptake [89].

The body weight change is an important parameter for safety assessment of endoradiotherapy. During ^225^Ac-TM TAT, no acute effects on the body weight were visible, underlining that the applied amount of activity (5 kBq per animal; approximately 200 kBq/kg) was safe and caused no considerable toxicities. According to biodistribution data with the ^64^Cu-TM, which showed no unspecific radiotracer accumulation, adverse reactions should be limited to the hematopoietic system. Consequently, one would expect that the ^225^Ac-TM should have less side effects than the ^225^Ac-conjugated PSMA ligand, which shows long-term toxicities and late radiation nephropathy as a dose-limiting toxicity [82,90,91,92]. Favorable safety profiles were also observed with other Ab-based, theranostic compounds. ^117^Lu-J591 [93,94] or ^225^Ac-J591 mAbs [95,96] show fewer side effects on kidneys, small intestine, and salivary glands but higher hematologic toxicity, which was manageable by fractionated drug administration [94].

## 5. Conclusions

Overall, our data show that the novel anti-PSCA IgG4-TM can be used multimodally for diagnostic and therapeutic purposes: (i) highly specific and efficient UniCAR T cell immunotherapy, (ii) in vivo molecular imaging with the ^64^Cu-TM, and (iii) PSCA-targeted radioimmunotherapy using the ^225^Ac-TM. Considering future perspectives, it is an excellent candidate for an image-guided, combinatorial treatment approach that is expected to have synergistic effects. Radiation is known to induce a pro-inflammatory TME, remodel the tumor vasculature, and enhance expression of chemoattractant targets, MHCI, and adhesion molecules [97,98,99]. This in turn improves tumor infiltration with both endogenous and genetically engineered immune effector cells [100], as well as soluble factors (e.g., theranostic TMs). Thus, local tumor irradiation by the ^225^Ac-TM could potentially counteract the immunosuppressive TME found in PCa and promote UniCAR T cell effectiveness. This assumption is supported by first preclinical studies demonstrating synergistic effects of radiotherapy and (CAR T cell) immunotherapy [100,101,102,103]. In terms of future clinical implementation, treatment-emergent imaging with the ^64^Cu-TM could enable more accurate treatment decisions leading to an individually tailored PCa management for each patient.

## Data Availability

The authors confirm that the data supporting the findings of this study are available within the article and its Supplementary Materials.

## Supplementary Materials

The following supporting information can be downloaded at: https://www.mdpi.com/article/10.3390/cancers14081996/s1, Figure S1: Uncropped Western Blot image.; Figure S2: Redirection of UniCAR T cells to LNCaP-PSCA cells via the novel anti-PSCA IgG4-TM.; Figure S3: Dual-targeting of LNCaP-PSCA cells using the novel anti-PSCA IgG4-TM and the PSMA-L TM.
