# Supplementary material for: Development and Functional Characterization of a Versatile Radio-/Immunotheranostic Tool for Prostate Cancer Management
Source: Cancers (Basel). 2022 Apr 14;14(8):1996. doi: 10.3390/cancers14081996 (PMC9027777; doi:10.3390/cancers14081996)
Supplement: Supplementary file 1 [file cancers-14-01996-s001.zip › cancers-1647365-supplementary.pdf]

cancers

MDPI

Supplementary Materials

Development and Functional Characterization of a Versatile Radio-/Immunotheranostic Tool for Prostate Cancer Management

Claudia Arndt [1,2], Ralf Bergmann [1,3], Franziska Striese [1], Keresztély Merkel [3,4], Domokos Máthé [3,5], Liliana R. Loureiro [1], Nicola Mitwasi [1], Alexandra Kegler [1], Frederick Fasslrinner [2,6], Karla Elizabeth González Soto [1], Christin Neuber [1], Nicole Berndt [1], Noemi Kovács [3], David Szöllősi [3], Nikolett Hegedűs [3], Gyula Tóth [7], Jan-Philipp Emmermann [3], Kuzhuvelil B. Harikumar [8], Tibor Kovacs [9], Michael Bachmann [1,10,11,12,*] and Anja Feldmann [1]

Helmholtz-Zentrum Dresden-Rossendorf, Institute of Radiopharmaceutical Cancer Research, D-01328 Dresden, Germany; c.arndt@hzdr.de (C.A.); r.bergmann@hzdr.de (R.B.); f.striese@rotop-pharmaka.de (F.S.); l.loureiro@hzdr.de (L.R.L.); n.mitwasi@hzdr.de (N.M.); a.kegler@hzdr.de (A.K.); k.gonzalez@hzdr.de (K.E.G.S.); c.neuber@hzdr.de (C.N.); n.berndt@hzdr.de (N.B.); a.feldmann@hzdr.de (A.F.)

[2] Mildred Scheel Early Career Center, Faculty of Medicine Carl Gustav Carus, TU Dresden, D-01328 Dresden, Germany; frederick.fasslrinner@uniklinikum-dresden.de

[3] Department of Biophysics and Radiation Biology, Faculty of Medicine, Semmelweis University, Tűzoltó u. 37-47, H-1094 Budapest, Hungary; merkel.keresztely@phd.semmelweis-univ.hu (K.M.); domokos.mathe@hcemm.eu (D.M.); noemi.kovacs@hcemm.eu (N.K.); szollosi.david@med.semmelweis-uni.hu (D.S.); hegedus.nikolett@med.semmelweis-univ.hu (N.H.); jan.emmermann@stud.semmelweis.hu (J.-P.E.)

[4] University No. 1 Clinic for Surgery, Faculty of Medicine, Semmelweis University, H-1094 Budapest, Hungary

[5] Hungarian Centre of Excellence for Molecular Medicine, In Vivo Imaging Advanced Core Facility, H-6723 Szeged, Hungary

[6] Medical Clinic and Polyclinic I, Medical Faculty, University Hospital Carl Gustav Carus, Technical University Dresden, D-01307 Dresden, Germany

[7] Department: Cyclotron & Radiochemistry, Pozitron Ltd., H-1094 Budapest, Hungary; gyula.toth@pet.hu

[8] Cancer Research Program, Rajiv Gandhi Centre for Biotechnology (RGCB), Thiruvananthapuram 695 014, Kerala, India; harikumar@rgcb.res.in

[9] Institute of Radiochemistry and Radioecology, University of Pannonia, H-8200 Veszprem, Hungary; kt@almos.uni-pannon.hu

[10] National Center for Tumor Diseases (NCT), D-01307 Dresden, Germany; German Cancer Research Center (DKFZ), D-69120 Heidelberg, Germany; Faculty of Medicine and University Hospital Carl Gustav Carus, Technische Universität Dresden, D-01307 Dresden, Germany; Helmholtz-Zentrum Dresden-Rossendorf (HZDR), D-01328 Dresden, Germany

[11] German Cancer Consortium (DKTK), Partner Site Dresden and German Cancer Research Center (DKFZ), D-69120 Heidelberg, Germany

[12] Tumor Immunology, University Cancer Center (UCC), University Hospital Carl Gustav Carus, Technical University Dresden, D-01307 Dresden, Germany

* Correspondence: m.bachmann@hzdr.de

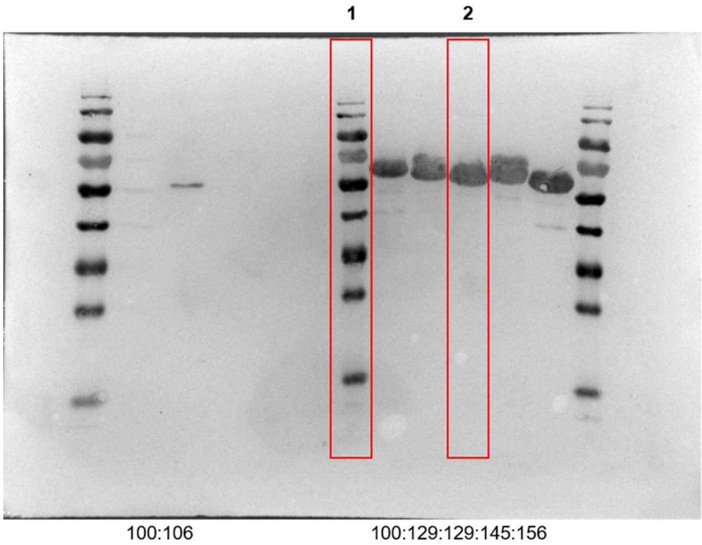

Figure S1. Uncropped Western Blot image. Uncropped Western Blot image corresponding to the two lanes (red boxes, lane 1 and 2) shown in Figure 2E of the manuscript. Lane 1: molecular weight marker; Lane 2: anti-PSCA IgG4-TM elution fraction. All the other lanes include molecules not related to the work developed in the submitted manuscript.

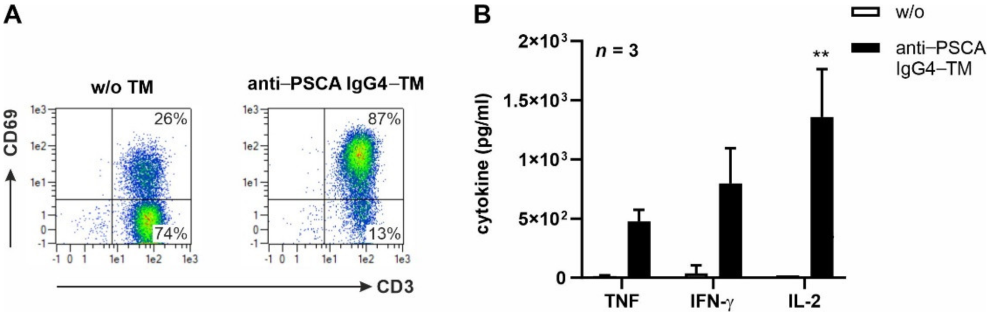

Figure S2. Redirection of UniCAR T cells to LNCaP-PSCA cells via the novel anti-PSCA IgG4-TM. UniCAR T cells were incubated with LNCaP-PSCA cells at an E:T ratio of 5:1 in the presence or absence of 5 nM TM. After 24 hours, **(A)** CD69 expression on UniCAR T cells and **(B)** secretion of TNF, IFN-γ and IL-2 were analyzed via flow cytometry or ELISA, respectively. **(B)** Summarized data of three different T cell donors are shown as mean ± SEM (** $p < 0.01$, compared to samples w/o the anti-PSCA IgG4-TM; two-way ANOVA with post-hoc Šídák's multiple comparisons test).

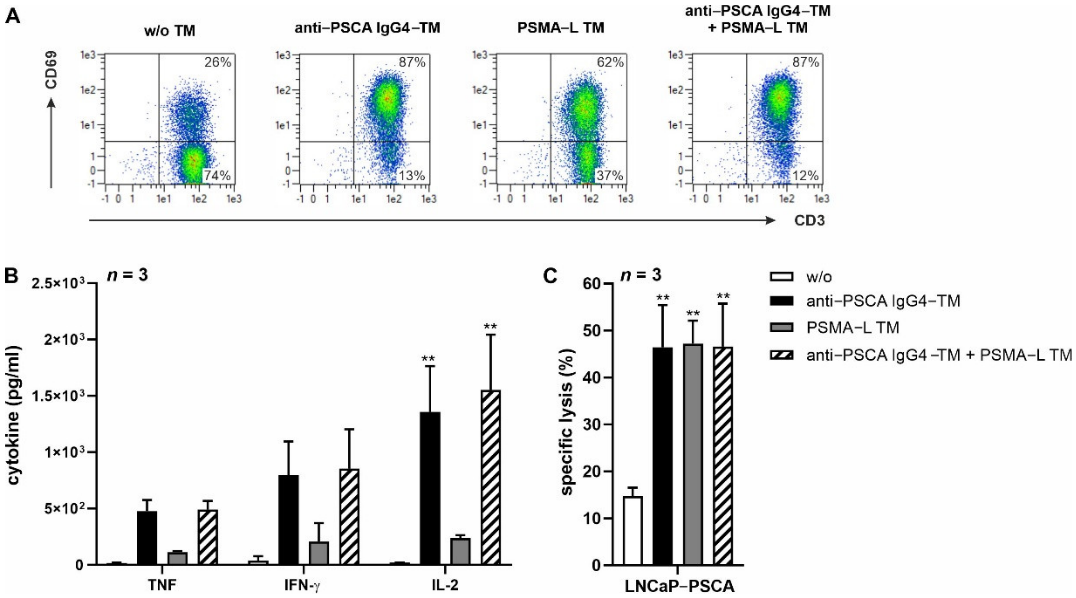

Figure S3. Dual-targeting of LNCaP-PSCA cells using the novel anti-PSCA IgG4-TM and the PSMA-L TM. UniCAR T cells were incubated with LNCaP-PSCA cells at an E:T ratio of 5:1 in the presence or absence of one or both TMs (5 nM each). After 24 hours, **(A)** CD69 expression on UniCAR T cells, **(B)** secretion of TNF, IFN-γ and IL-2, and **(C)** tumor cell killing were analyzed via flow cytometry, ELISA or standard chromium release assay, respectively. **(B,C)** Summarized data of three different T cell donors are shown as mean ± SEM (** $p < 0.01$, compared to samples w/o TM; two-way ANOVA with post-hoc Šídák's multiple comparisons test).
